# What Can Happen When Postpartum Anxiety Progresses to Psychosis? A Case Study

**DOI:** 10.1155/2018/8262043

**Published:** 2018-02-20

**Authors:** Vesna Pirec

**Affiliations:** ^1^Eating Recovery Center/Insight in Chicago, 333 North Michigan Ave, Suite 1900, Chicago, IL 60601, USA; ^2^Department of Psychiatry, University of Illinois at Chicago, 921 S. Wood, Chicago, IL 60612, USA

## Abstract

This case report describes a primipara without documented psychiatric history prior to complicated delivery. Onset of severe insomnia and anxiety was right after childbirth but not treated. Obsessive thinking pattern became more prominent. The patient became depressed and sought psychiatric help four months after delivery. Insomnia was then treated pharmacologically. Anxiety and depression persisted, suicidal ideation emerged, and the patient became confused, indecisive, overwhelmed, and delusional regarding her child's health. Medications for depression and anxiety were started six months postpartum yet were ineffective. The patient's obsessions gradually became fully psychotic and she committed an altruistic infanticide eight months postpartum. Psychiatric hospitalization occurred, followed by a long course of mental, physical, legal, and social rehabilitation. She was minimally responsive to psychopharmacological treatment, which appeared to be partly related to her hormonal dysregulation. Several months into the treatment she gradually started improving and returned to baseline two years later. The Illinois court found the patient not guilty to murder by reason of Insanity.

## 1. Introduction

Postpartum psychiatric disorders are fairly common and still pose a great risk for mother, child, and the entire family [1]. The spectrum of potential consequences is wide, from lack of the attention to the baby with consequences related to the attachment and baby's development, as well as the child's mental health, to risks of suicide and infanticide.

While postpartum depression has been more discussed and better recognized [2], postpartum anxiety is still less commonly addressed. That is in particular true in new mothers without previously identified psychiatric diagnosis. Furthermore, not much is known nor discussed about postpartum anxiety where intrusive thoughts are dominant and often coupled with severe insomnia, initially without any other comorbid depressive symptoms. When not addressed and treated these initially relatively benign symptoms can develop into psychosis that clinically presents differently than postpartum psychosis associated with bipolar disorder.

Here we present a case of a woman with no previous psychiatric history who developed symptoms of severe postpartum anxiety, insomnia, and consequently depression. While the patient's anxieties were mainly interpreted as parts of her personality characteristics and normalized, her development of psychosis was not fully recognized leading into murdering her eight-month-old child.

## 2. Case Report

The patient described was a 30-year-old married, employed, and domiciled woman, primipara, with no previously documented psychiatric history. Family history was significant for mild depression in both parents. There was no family perinatal illness documented. Patient delivered a healthy baby via Caesarian section after a prolonged and traumatic labor due to the baby's breech position. During her pregnancy, the patient dealt with minor anxiety symptoms that worsened over the course of the pregnancy linked to “too much planning.”

The patient reported symptoms of postpartum blues immediately after the delivery. During the first three to four months of the child's life, the patient was the main caregiver. At times, she was indecisive and her parenting style was somewhat rigid with constant attempts to follow strict schedules and guidelines. Insomnia, which predated her pregnancy, became more prominent after the delivery. The patient lost 27 kg within two months postpartum. Three months postpartum anxiety and worrisome thinking patterns became prominent in anticipation of returning to work. Despite the support provided by her family, the patient constantly felt overwhelmed. Initial and midnight insomnia were severe. She started having difficulty managing her responsibilities at both home and work. After breastfeeding for four months the patient lost her milk supply. Led by guilt, she attributed formula feeding to “developmental issues.” She was convinced that “they did everything wrong.” The baby was evaluated by a developmental specialist, but no major issues were found.

Approximately five months postpartum, the patient started having severe concerns regarding her baby's health and development. She was convinced that the baby was “shrinking” and “not growing out of clothes as appropriate” and believed that the baby's skin “was grey” and that something was wrong with its neck. She shared concerns with the pediatrician and was reassured. Patient's insomnia and anxiety worsened leading to severe depression five months postpartum. She started attending weekly psychotherapy. Despite treatment, suicidal thoughts emerged, one with a plan to jump from a friend's balcony of a high rise. Family minimized complaints and sought no immediate professional assistance. The patient continued deteriorating and had more difficulties taking care of herself and the baby. This episode was followed by a period of ten days during which the patient barely slept. In an attempt to get some rest, she took ten zolpidem pills (prescribed by her obstetrician). This was interpreted as a suicidal gesture which led to her first psychiatric hospitalization. There the patient was diagnosed with adjustment disorder and insomnia and discharged without any medications. Due to concerns regarding the patient's deterioration and depressive symptoms, the family took her to another hospital for a reevaluation. She was admitted again and diagnosed with Major Depressive Disorder. Mirtazapine (15 mg at bedtime) was introduced to treat depression and insomnia. After brief hospital stay the patient was referred to an outside psychiatrist yet continued to deteriorate despite medication adjustment. The patient was convinced that her child was suffering due to “malformations” and “inadequate development.” She withdrew from her family and surroundings and was minimally responsive, both emotionally and verbally. She lost her job. Paranoid thoughts emerged along with responses to inner stimuli. For the most part, her family took over caring for the child. Mirtazapine was discontinued and escitalopram started. Due to her paranoia worsening, her psychiatrist added olanzapine 5 mg daily to her regimen. The patient became overly sedated and discontinued olanzapine on her own after couple of days. After that her mood suddenly improved, and she became more energized and started participating in everyday activities. Two days later, while unsupervised with the child, the patient smothered the 8-month-old baby. After the incident, the patient was admitted to a psychiatric hospital for two weeks, after which she was followed by our institution.

With us the patient initially presented as catatonic, with blunted affect, and minimally verbal, and appeared psychotic, while responding to inner stimuli. She was confused and kept asking about her baby, not remembering the period around the day of infanticide. When admitted the patient was already taking citalopram 60 mg daily and risperidone 2.5 mg daily but was poorly responsive and remained confused, amnesic, severely depressed, and emotionally unresponsive. The patient's affect was restricted, and periods of intense blinking were observed. She often presented as disheveled with poor hygiene. Due to treatment resistance and severity of symptoms, electroconvulsive treatment (ECT) was suggested but the patient and family refused. Three months after cessation of breastfeeding the patient's prolactin level continued to be elevated, secondary amenorrhea persisted, and she developed facial cystic acne. Her thyroid function was within normal limits. Gonadal hormone levels were slightly decreased. Medications were adjusted and risperidone (2.5 mg daily) was cross tapered to ziprasidone (up to 120 mg daily in divided doses). Lorazepam was added, which initially helped with insomnia and anxiety. Over the course of four weeks of hospital treatment, the patient's prolactin level dropped from 135.5 to 22.5 ng/ml (still elevated) and yet her period had not restarted. After five weeks of inpatient treatment at our facility, the patient gradually improved; however minimal emotional reactivity was observed. Cognitive cloudiness lifted. Upon seeing her baby's photos at that point, she stated that it was not how she saw or remembered her child.

Following the hospitalization, the patient continued treatment in the partial hospitalization program for three months. Individual psychotherapy and psychiatry appointments continued beyond. Depression, anxiety, and insomnia persisted for over a year after the incident, but psychotic symptoms subsided. Six months after the incident the patient regained memory of the night her child died. The patient's amenorrhea persisted eight months after cessation of breastfeeding, despite prolactin levels normalizing six months postpartum, reaching a low point of 1.7 ng/ml. Estrogen levels were low. Cystic facial acne did not respond to antibiotic treatment. She was started on progesterone challenge by gynecologist for two weeks, fourteen months postpartum, without any improvement. Oral contraceptive pills (OCP) were prescribed subsequently to progesterone challenge. While on OCP the patient had breakthrough bleeding and acne persisted. She was continuously emotionally numb, with slow cognitive processing. The patient now reconsidered ECT. In preparation for ECT brain magnetic resonance imaging (MRI) demonstrated pituitary fossa asymmetry and lacunar infarctions in basal ganglia. The consulting neurologist was not concerned about the findings and could not directly link them to the patient's clinical presentation. However, the patient decided against ECT and opted for medication adjustment. Ziprasidone was gradually discontinued and aripiprazole was started 17 months postpartum to augment antidepressants. This was minimally helpful. Zolpidem or lorazepam with Benadryl was periodically used to treat her insomnia. Due to severe weigh gain, aripiprazole was gradually discontinued. Eventually, the patient discontinued her OCP and started menstruating spontaneously 21 months postpartum. At that time her symptoms started to remit, acne was no longer present, and her mood and functionality ameliorated.

Two weeks after the incident the patient was charged with first-degree murder. A year and a half after the baby's death, the case was taken to the court and the patient was acquitted of charges based on Insanity at the time of the act. She was court mandated to continue treatment with a psychotherapist and a psychiatrist. She got separated and then divorced approximately eighteen months after the incident. It took more than two and a half years of intense and regular outpatient treatment for the patient to return to her baseline while being on a maintenance dose of 20 mg citalopram daily.

## 3. Discussion

Screening for postpartum depression (PPD) is mandated in many countries and in at least five states within the US [3]. Screening is usually done by administering a brief self-assessment tool such as Edinburgh's postpartum depression scale (EPDS) [4] or Patient Health Questionnaire 9 (PHQ9) [5, 6]. These measures improved recognition and appropriate referral to treatment for some women [7–9], yet still leaving many behind. A shortcoming of the current approach is that screening is not conducted beyond six weeks postpartum where some women do not develop severe symptoms during that timeframe [10]. Additionally, neither of the tools used detects symptoms of perinatal anxiety which may be more common [11] and could even precede postpartum depression (such as in this case). Thus, women with no prior psychiatric conditions and therefore regular follow-up by psychologist/psychiatrist could be easily missed and not adequately treated. Providers that do see new moms, such as pediatricians, mostly focus their attention on the baby. Concerns brought up by new mothers tend to be normalized by a health care team, without further assessment or evaluation of mother's issues. This current medical approach leaves vulnerable women to rely on themselves and their families, who often do not comprehend the severity of these symptoms. Both of these scenarios occurred in the presented case. The severity of this patient's concerns regarding her child's health and wellbeing was not fully evaluated leaving her unsupported. Additionally, worsening of the patient's unaddressed anxiety and depression led to more severe symptoms and inability to adequately express her feelings and concerns.

Postpartum anxiety is common [12]. While many new mothers tend to worry about their child's safety, development, and health, at times those thoughts become more intrusive and obsessive in nature. Women may believe that they have done something wrong or will somehow harm the baby. In order to control these thoughts, rigid rituals and structure are developed along with compulsive checking on the child. However, if a woman feels that she is losing control, such as when this patient needed to handle the care to other family members, anxiety may progress dramatically.

The continuum from full insight via intrusive thoughts to delusional thinking has been described and debated [13] but is still poorly understood. These phenomena have been recognized in both peripartum and nonperipartum populations, etiologies of which may differ.

Insomnia has been one of the potential triggers for both anxiety [14, 15] and psychosis [16, 17]. Several studies correlated insomnia in pregnancy and postpartum with higher risk of postpartum depression [18, 19].

Insomnia has been documented to be a prominent symptom in postpartum mood, anxiety, and psychosis with prevalence rates reported between 42% and 100% [20–23]. In this case, severe insomnia predated pregnancy, becoming significantly worse postpartum and as anxiety symptoms progressed, culminating in ten days without sleep and suicidal gestures in an attempt to “get rest.” Worsening of postpartum insomnia has been linked with several hormonal theories. Oxytocin inducing labor and later on breastfeeding has been called a “wake hormone” [24]. Furthermore, cessation of breastfeeding and decrease in oxytocin could worsen depression and anxiety [25]. In this case, cessation of breastfeeding was abrupt and most likely linked to severe anxiety. That was the period in which obsessional intrusive thoughts started transforming into delusional thinking, progressively leading to paranoia and psychosis. At admission to our unit, the patient's prolactin level was highly elevated, which may have resulted from exposure to antipsychotic medication or other causes. However, increased prolactin levels have been reported to trigger psychosis in nonpregnant samples [26]. The relationship between hyperprolactinemia and psychosis in our case is hard to determine due to other contributing factors. Additionally, even after the prolactin level dropped, hypogonadism, amenorrhea, and acne persisted, along with significant cognitive slowing, severe memory impairment, and persisting delusions related to the child's health status prior to his death. Furthermore, abrupt drops in progesterone and estrogen upon delivery have been shown to adversely affect serotonergic functioning and to increase vulnerability to anxiety [27, 28]. Progesterone challenge and oral contraceptive treatment did not ameliorate the patient's hormonal or cognitive functioning. Nonspecific changes in pituitary fossa and basal ganglia described on her MRI could have also been related to patient's hormonal imbalance [29]. No conclusion can be drawn relative to the impact of hormonal status in this patient's presentation and her relative resistance to psychotropic medications. However, it appears that regaining hormonal balance and the onset of regular periods lead to her recovery. Some of the mentally ill patients who commit infanticide are psychotic [30, 31]. Postpartum psychosis present more abruptly after the delivery is easier recognized and considered a psychiatric emergency [32]. Up to four percent of women with postpartum psychosis commit infanticide [33, 34]. Subtle symptom development, such as in this case, may be difficult to detect, leading to potentially more fatalities.

While what triggered the symptom progression in this case cannot be determined, it appears that the combination of significant postpartum anxiety, insomnia, and persistent hormonal imbalance, while not recognized or treated, contributed to the psychopathology. This case underscores the importance of more careful assessment and evaluation of mothers with no prior psychiatric diagnosis and appropriate treatment that could prevent drastic consequences.
